# Macrolide derivatives reduce proinflammatory macrophage activation and macrophage‐mediated neurotoxicity

**DOI:** 10.1111/cns.13092

**Published:** 2019-01-24

**Authors:** Bei Zhang, Timothy J. Kopper, Xiaodong Liu, Zheng Cui, Steven G. Van Lanen, John C. Gensel

**Affiliations:** ^1^ Department of Physiology, College of Medicine, Spinal Cord and Brain Injury Research Center University of Kentucky Lexington Kentucky; ^2^ Division of Bioorganic, Medicinal, & Computational Chemistry, College of Pharmacy University of Kentucky Lexington Kentucky

**Keywords:** brain, erythromycin, M2, microglia, spinal cord injury, stroke

## Abstract

**Introduction:**

Azithromycin (AZM) and other macrolide antibiotics are applied as immunomodulatory treatments for CNS disorders. The immunomodulatory and antibiotic properties of AZM are purportedly independent.

**Aims:**

To improve the efficacy and reduce antibiotic resistance risk of AZM‐based therapies, we evaluated the immunomodulatory and neuroprotective properties of novel AZM derivatives. We semisynthetically prepared derivatives by altering sugar moieties established as important for inhibiting bacterial protein synthesis. Bone marrow‐derived macrophages (BMDMs) were stimulated in vitro with proinflammatory, M1, stimuli (LPS + INF‐gamma) with and without derivative costimulation. Pro‐ and anti‐inflammatory cytokine production, IL‐12 and IL‐10, respectively, was quantified using ELISA. Neuron culture treatment with BMDM supernatant was used to assess derivative neuroprotective potential.

**Results:**

Azithromycin and some derivatives increased IL‐10 and reduced IL‐12 production of M1 macrophages. IL‐10/IL‐12 cytokine shifts closely correlated with the ability of AZM and derivatives to mitigate macrophage neurotoxicity.

**Conclusions:**

Sugar moieties that bind bacterial ribosomal complexes can be modified in a manner that retains AZM immunomodulation and neuroprotection. Since the effects of BMDMs in vitro are predictive of CNS macrophage responses, our results open new therapeutic avenues for managing maladaptive CNS inflammation and support utilization of IL‐10/12 cytokine profiles as indicators of macrophage polarization and neurotoxicity.

## 1 INTRODUCTION

The management of maladaptive inflammation is an emerging therapeutic target for many neuropathologies. Different macrophage phenotypes have been identified in the injured central nervous system (CNS) in conditions such as ischemic brain damage, spinal cord injury, and traumatic brain injury. After spinal cord injury, for example, there is a heterogeneous neuroinflammatory response mediated by resident microglia and infiltrating macrophages. Classically activated macrophages (M1) secrete proinflammatory cytokines and chemokines and contribute to continued cell death and a persistent inflammatory microenvironment within the injured spinal cord.^1^, ^2^ In contrast, alternatively activated macrophages (M2) release anti‐inflammatory cytokines and facilitate tissue repair.^2^ Increasingly, clinicians and researcher are testing the therapeutic potential of drugs that polarize macrophage activation toward reparative phenotypes in a variety of CNS disorders.

Macrolide antibiotics are a class of natural products consisting of a highly substituted macrocyclic 14‐, 15‐, or 16‐membered lactone ring. Azithromycin (AZM) is a 15‐membered, second generation, synthetic derivative of erythromycin with improved pharmacokinetic properties and a broad antimicrobial spectrum.^3^ Azithromycin is well tolerated and commonly prescribed. Moreover, AZM becomes highly concentrated in macrophages and other phagocytes.^4^, ^5^ Across a variety of inflammatory conditions, AZM attenuates proinflammatory cytokine production by macrophages and other immune cells.^6^

Azithromycin and other macrolide antibiotics are now being tested as immunomodulatory agents for CNS disorders. Specifically, we and others observed immunomodulatory effects and improved recovery with AZM treatment in spinal cord injury, stroke, and retinal ischemia/reperfusion injury.^7^, ^8^, ^9^, ^10^, ^11^, ^12^ The neuroprotective properties of AZM in these models are associated with direct effects on macrophages.^7^, ^8^, ^9^ We have shown that in vitro application of AZM to proinflammatory M1 bone marrow‐derived macrophages (BMDMs) dampens the release of proinflammatory cytokines, increases M2‐associated anti‐inflammatory cytokines, and reduces the neurotoxicity of M1 macrophage‐conditioned medium.^7^

In efforts to improve efficacy and/or reduce the risk of increasing antibiotic resistance, researchers are evaluating the immunomodulatory potential of AZM derivatives and other macrolide derivatives with the goal of separating the antibiotic from immunomodulatory properties. As a result, some macrolide derivatives have been shown to retain immunomodulatory properties in models of lung inflammation, inflammatory bowel diseases, arthritis, and skin inflammation.^13^, ^14^, ^15^, ^16^, ^17^, ^18^ The ability of macrolide derivatives to reduce macrophage‐mediated neurotoxicity, however, is unknown. With the increased use of AZM as an immunomodulatory agent for macrophage‐mediated neurotoxicity in CNS pathologies, our goal in the present study was to determine whether macrolide derivatives retain neuroprotective properties. Using a semisynthetic approach to target modification of the sugar moieties of AZM, we generated a small library of derivatives, some of which lacked the cladinose found in the parent. We then tested the cytokine profiles and neurotoxicity of M1‐stimulated BMDMs treated with derivatives and observed that unique derivatives reduce M1‐macrophage activation and subsequent neuron death. Previously we determined that the effect of BMDMs in vitro is predictive of macrophage responses in the injured CNS^8^, ^19^, ^20^; therefore, the results of the current study open new therapeutic avenues for the management of maladaptive inflammation in CNS disorders.

## 2 METHODS

### 2.1 Semisynthesis of AZM derivatives

Like other macrolides, AZM inhibits bacterial protein synthesis by binding to the 50S subunit of ribosome and thus interfering with the growth of the polypeptide chain.^21^, ^22^ Specifically, the sugar moieties are known to play an essential role in establishing binding interactions with the bacterial ribosomal assembly. We used a semisynthetic approach to create derivatives with targeted alterations in these bacterial binding sugar residues. The AZM derivatives that were synthesized and structurally confirmed by spectroscopic analysis are listed in Table 1. See Supporting information for mass and H NMR spectrum profiles.

**Table 1:** Structure, molecular weight, and antibiotic properties of derivatives

Derivatives name	Structure	Molecular weight	MIC^a^ (μmol/L)
AZM (parent compound)	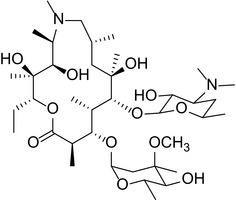	748.51	1.0
AZM1	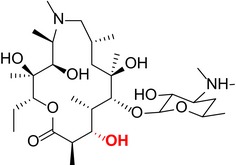	590.79	1000
AZM4	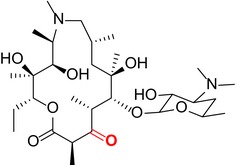	588.77	500
AZM5	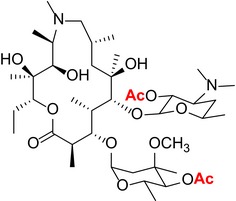	833.06	2.0
AZM7	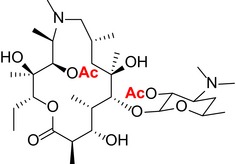	674.44	1000

a Minimum inhibitory concentration against *Staphylococcus aureus* subsp aureus Rosenbach (ATCC 6538).

AZM 1: The removal of the cladinose of AZM followed a previously described procedure.^23^ Briefly, AZM (6.67 mmol) was dissolved into 200 mL of methanol containing 1% of conc. HCl, and the solution was stirred at room temperature for 24 hours Saturated NaHCO_3_ solution was added to neutralilze the solution, and the solvent was removed under vacuum. The product was purified by flash column chromatography with silica gel (EtOAc/MeOH) to give AZM1 (5.53 mmol). MS (ESI) *m/z*: [*M* + H]^+^ calcd for C_30_H_59_N_2_O_9_ 591.4; found 591.4.

AZM4: AZM1 (4.00 mmol) was dissolved into 100 mL anhydrous ethyl acetate. Acetic anhydride (3.67 mL, 40.0 mmol) was added, and the solution was stirred for 2 hours at room temperature. The mixture was washed twice with NaHCO_3_ (5% in water, 30 mL), and the organic phase was dried under vacuum. The product was purified by flash column chromatography with silica gel (DCM/MeOH) to give AZM2 (3.22 mmol). AZM2 (2.0 mmol) was dissolved into 40 mL anhydrous acetone solution on ice, and 2.0 mL Jones reagent was added dropwise. After 30 minutes, 10 mL of methanol and 10 mL saturated NaHCO_3_ solutions were added and the solid precipitate was collected by filtration and dried under vacuum. The product was purified by flash column chromatography with silica gel (DCM/MeOH) to give AZM3 (1.30 mmol). AZM3 (1.0 mmol) was dissolved into 30 mL methanol, and sodium methoxide (5.0 mmol) with 5 mL of water was added. The solution was stirred at room temperature for 3 hours, and the solvent was removed under vacuum. The product was purified by flash column chromatography with silica gel (DCM/MeOH) to give 0.44 g AZM4 (0.75 mmol). ^1^H NMR (400 MHz, Methanol‐*d*_4_) δ 5.47 (s, 1H), 4.98 (dd, *J* = 9.8, 3.0 Hz, 1H), 4.16 (d, *J* = 7.4 Hz, 1H), 3.78 (d, *J* = 9.9 Hz, 1H), 3.65 (s, 1H), 3.55 (ddd, *J* = 11.0, 6.1, 1.9 Hz, 2H), 3.30‐3.23 (m, 1H), 3.02 (d, *J* = 12.3 Hz, 1H), 2.79 (q, *J* = 7.1 Hz, 1H), 2.60 (ddd, *J* = 12.2, 10.2, 4.2 Hz, 1H), 2.32 (s, 6H), 2.22 (d, *J* = 2.2 Hz, 3H), 2.20 (d, *J* = 2.9 Hz, 1H), 1.98 (d, *J* = 10.6 Hz, 1H), 1.85 (ddd, *J* = 11.5, 7.5, 3.9 Hz, 3H), 1.72 (ddd, *J* = 12.8, 4.2, 2.0 Hz, 2H), 1.46 (d, *J* = 14.7 Hz, 1H), 1.37 (s, 3H), 1.33‐1.26 (m, 2H), 1.24 (d, *J* = 4.5 Hz, 1H), 1.19 (td, *J* = 10.5, 10.1, 7.2 Hz, 9H), 1.07 (d, *J* = 6.7 Hz, 5H), 1.00 (d, *J* = 6.6 Hz, 3H), 0.87 (dd, *J* = 8.4, 6.5 Hz, 3H). ^13^C NMR (101 MHz, Methanol‐*d*_4_) δ 174.43, 106.50, 104.20, 93.68, 82.68, 70.25, 68.82, 64.29, 64.17, 61.12, 53.40, 48.44, 45.18, 43.74, 39.56, 30.86, 28.80, 25.39, 20.08, 20.04, 16.14, 15.22, 11.01, 10.87, 9.78, 9.42, 7.46. MS (ESI) *m/z*: [*M* + H]^+^ calcd for C_30_H_57_N_2_O_9_ 589.4; found 589.4.

AZM5: AZM (2.0 mmol) was dissolved into 50 mL anhydrous dimethylene chloride. Acetic anhydride (1.88 mL, 20.0 mmol) was added, and the solution was stirred for 1 hour The mixture was washed twice with NaHCO_3_ (5% in water, 30 mL), and the organic phase was dried under vacuum. The product was purified by flash column chromatography with silica gel (EtOAc/MeOH) to give 1.16 g AZM5 (1.39 mmol). ^1^H NMR (400 MHz, Methanol‐*d*_4_) δ 5.48 (s, 0H), 5.09 (d, *J* = 4.7 Hz, 1H), 5.00 (dd, *J* = 10.4, 2.5 Hz, 1H), 4.97 (d, *J* = 4.7 Hz, 1H), 4.71 (d, *J* = 4.3 Hz, 1H), 4.69‐4.61 (m, 2H), 4.42‐4.30 (m, 2H), 4.16 (dd, *J* = 5.0, 1.9 Hz, 1H), 4.10 (dd, *J* = 6.8, 2.5 Hz, 1H), 3.94‐3.77 (m, 2H), 3.62 (dd, *J* = 6.4, 3.1 Hz, 1H), 3.59 (d, *J* = 1.9 Hz, 1H), 3.42 (ddd, *J* = 10.7, 7.2, 3.6 Hz, 1H), 3.36 (d, *J* = 10.2 Hz, 3H), 3.27‐3.14 (m, 2H), 2.96‐2.89 (m, 1H), 2.89‐2.82 (m, 1H), 2.73 (d, *J* = 3.5 Hz, 6H), 2.49 (d, *J* = 15.3 Hz, 1H), 2.08 (s, 3H), 2.05‐1.95 (m, 3H), 1.89 (s, 6H), 1.84 (ddd, *J* = 7.9, 6.0, 2.3 Hz, 1H), 1.79‐1.63 (m, 3H), 1.56‐1.40 (m, 4H), 1.34 (d, *J* = 29.4 Hz, 3H), 1.28‐1.20 (m, 9H), 1.16 (t, *J* = 6.6 Hz, 3H), 1.14‐1.09 (m, 6H), 1.07 (d, *J* = 7.5 Hz, 3H), 0.99 (dd, *J* = 13.7, 6.9 Hz, 3H), 0.88 (q, *J* = 7.5 Hz, 3H). ^13^C NMR (101 MHz, Methanol‐*d*_4_) δ 178.13, 170.68, 170.67, 101.68, 101.59, 96.21, 95.13, 76.53, 74.67, 74.56, 74.24, 73.79, 72.99, 72.95, 69.64, 67.15, 66.73, 65.33, 65.12, 64.63, 63.05, 62.94, 48.75, 48.73, 45.13, 41.78, 38.72, 35.52, 30.25, 25.45, 25.31, 22.41, 20.73, 20.58, 20.35, 20.14, 19.44, 19.39, 17.08, 16.96, 15.96, 14.10, 10.09, 10.08. MS (ESI) *m/z*: [*M* + H]^+^ calcd for C_42_H_77_N_2_O_14_ 834.5, found 834.5.

AZM7: AZM (2.0 mmol) was dissolved into 80 mL anhydrous ethyl acetate. 3.76 mL (40.0 mmol) of acetic anhydride and 4‐dimethylaminopyridine (0.20 mmol) were added. After stirring at room temperature for 12 hours, the mixture was washed twice with NaHCO_3_ (5% in water, 30 mL), and the organic phase was dried under vacuum. The product was purified by flash column chromatography with silica gel (DCM/MeOH) to give AZM6 (1.64 mmol). AZM6 (1.0 mmol) was dissolved into 40 mL of methanol containing 1% of conc. HCl, and the solution was stirred at room temperature for 16 hours Saturated NaHCO_3_ solution was added to neutralilze the solution, and the solvent was removed under vacuum. The product was purified by flash column chromatography on silica gel (EtOAc/MeOH) to give 0.34 g AZM7. ^1^H NMR (400 MHz, Chloroform‐*d*) δ 4.96 (d, *J* = 5.6 Hz, 1H), 4.85 (dd, *J* = 10.6, 2.6 Hz, 1H), 4.79 (dd, *J* = 10.5, 7.6 Hz, 1H), 4.75‐4.67 (m, 1H), 4.62‐4.55 (m, 1H), 4.45 (dd, *J* = 9.7, 2.0 Hz, 1H), 3.88 (s, 1H), 3.66 (d, *J* = 2.1 Hz, 1H), 3.60‐3.52 (m, 2H), 3.22 (s, 3H), 3.18 (s, 1H), 2.94 (t, *J* = 9.6 Hz, 1H), 2.69 (dddt, *J* = 14.3, 11.0, 6.9, 3.9 Hz, 2H), 2.57 (t, *J* = 7.1 Hz, 1H), 2.37 (s, 1H), 2.23 (s, 6H), 2.09 (s, 3H), 2.06 (s, 3H), 2.02 (s, 3H), 1.82‐1.76 (m, 2H), 1.76‐1.68 (m, 2H), 1.38‐1.31 (m, 2H), 1.30‐1.24 (m, 6H), 1.24‐1.20 (m, 6H), 1.19 (d, *J* = 5.2 Hz, 4H), 0.98 (d, *J* = 6.7 Hz, 3H), 0.94 (d, *J* = 6.9 Hz, 2H), 0.91 (d, *J* = 7.2 Hz, 2H), 0.85 (t, *J* = 7.4 Hz, 3H). ^13^C NMR (101 MHz, Chloroform‐*d*) δ 169.96, 109.99, 101.25, 99.81, 98.81, 97.54, 78.04, 76.36, 75.46, 74.90, 73.96, 71.46, 70.85, 69.36, 64.44, 63.32, 60.36, 56.28, 49.01, 40.63, 37.75, 30.45, 26.15, 25.29, 21.37, 21.11, 21.01, 20.96, 20.91, 20.43, 18.17, 14.16. MS (ESI) *m/z*: [*M* + H]^+^ calcd for C_34_H_63_N_2_O_11_ 675.4, found 675.4.

### 2.2 Antibiotic activity

The minimum inhibitory concentration (MIC) displays the relative antibiotic potency of AZM and each derivative against a common strain of bacteria (*Staphylococcus aureus)*. The protocol used for the determination of the MIC was as previously described with minor modifications.^24^ *Staphylococcus aureus* subsp aureus Rosenbach (ATCC 6538) were grown in 5 mL of Bacto^TM^ Tryptic Soy Broth medium for 16 hours at 37°C with shaking (250 rpm) when an aliquot was diluted X1000 into 4.5 mL of fresh medium and incubated until OD_600_ reaching 0.4. An aliquot of the suspension was diluted X1000. An aliquot (90 μL) was transferred into an individual well of a 96‐well plate supplied with 5 μL of the test compound or control (AZM). Maximum final concentration of 1000 μmol/L with serial dilutions was used to measure the antistaphylococcal activity in comparison with the negative control (1% DMSO). The culture plates were incubated at 37°C for 16 hours with shaking (50 rpm). The OD_600_ of each well measured using BioTek™ Synergy™ (Biotek, Winooski, VT) 2 Multi‐Mode Microplate Readers. The acquired OD_600_ values were normalized to the negative control wells (100% viability). Resazurin solution (5 μL) was also added to each well, and the plates were shaken for 10 seconds and incubated at 37°C for another 3 hours to allow resazurin to convert to resorufin by viable bacteria. The minimal concentration of the tested compound that caused growth inhibition was recorded. The calculated MICs are shown in Table 1.

### 2.3 Preparation of bone marrow‐derived macrophages (BMDMs) and macrophage‐conditioned medium (MCM)

BMDMs were isolated from the femurs and tibias of C57BL/6 mice at 10‐16 weeks of age. In a tissue culture hood, the bones were flushed with a syringe filled with cold washing media (RPMI 1640 supplemented with 10% FBS and 1% penicillin/streptomycin) to extrude bone marrow into a sterile falcon tube. The bone marrow was then triturated three times using syringes fit with 18 gauge needles and then centrifuged at 1000 rpm for 5 minutes at 4°C. After removing supernatant, red blood cells were lysed in lysis buffer (0.15 mol/L NH4Cl, 10 mmol/L KHCO3, and 0.1 mmol/L Na2EDTA, pH 7.4) for 3 min. The remaining cells were washed once in washing media and then centrifuged at 212 *g* for 5 minutes at 4°C. The resulting cell pellet was resuspended in BMDM culture media (RPMI 1640 supplemented with 1% penicillin/streptomycin, 1% HEPES, 0.001% β‐mercaptoethanol, 10% FBS, and 20% supernatant from sL929 cells) and then plated in T75 flasks at a density of 1 × 10^6 ^cells/mL. The sL929 cell supernatant (cells, a generous gift from Phillip Popovich at The Ohio State University) contains macrophage colony stimulating factor, which is needed to promote differentiation of bone marrow cells into macrophages.^25^ Cell culture media was changed on days 2, 4, and 6, and then, cells were replated at the density of 1 × 10^6 ^cells/mL on day 7 for designated stimulation and/or azithromycin (AZM) treatment. The following day, BMDMs were stimulated to be M1 using LPS (50 ng/mL; Invivogen) plus IFN‐gamma (20 ng/mL; eBioscience) diluted in N2A growth medium as previously described.^7^ AZM (Sigma PHR1088) or AZM derivatives were diluted to the concentrations of 1, 5, 25, and 125 μmol/L and then added to the BMDMs at the time of stimulation. Unstimulated BMDMs maintained in N2A growth medium were used as control. Six hours following incubation, the supernatant of the stimulated macrophages (macrophage‐conditioned media (MCM)) was collected and centrifuged to remove the cell debris. The resulting media was either applied to Neuro‐2a cells for the measurement of neurotoxicity or tested for IL‐10 and IL‐12p40 levels using standard ELISA kits (Thermo Scientific, Rockford, IL).

### 2.4 Assessment of macrophage and neuron cell viability

BMDMs seeded in 96‐well plates (1 × 10^6 ^cells/mL) were treated with a range of concentrations (1‐125 μmol/L) of AZM or AZM derivatives for 24 hours Neuro2a (N2a) cell line is a generous gift from Chris Richards at the University of Kentucky. Cells were cultured in N2a growth media, which contains 45% DMEM and 45% Opti‐MEM Reduced‐Serum Medium (Life Technologies) supplemented with 10% FBS and 1% penicillin/streptomycin.^26^ Experiments were carried out using N2a cells within 12 passages. To test the neurotoxicity of MCM, N2a cells were seeded in 96‐well plates at a density of 2 × 10^5^ cells/mL for 24 hours in the growth media and then incubated in MCM for another 24 hours The cell viability of BMDMs or N2a cells was measured by using MTT assay according to the manufacturer instructions and as described previously (Sigma‐Aldrich).^7^

### 2.5 Statistical analyses

Results are expressed as mean ± standard deviation (SD) and analyzed using GraphPad Prism 6.0 (GraphPad Software). Data were compared by one‐way analysis of variance (ANOVA) among groups followed by Dunnett's or Holmes‐Sidak multiple comparison tests. Differences were determined to be statistically significant at *P* value ≤0.05.

## 3 RESULTS

### 3.1 Assessment of macrophage viability

**Figure 1:**
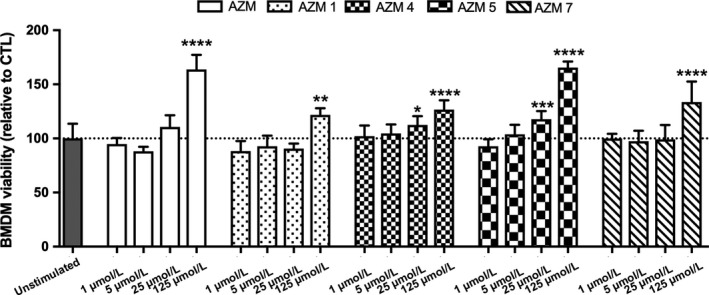
Altering the antibiotic properties of azithromycin does not decrease macrophage viability. Bone marrow‐derived macrophages (BMDMs) were isolated from adult mice and were treated with AZM, AZM1, AZM4, AZM5, and AZM7 at concentrations of 1, 5, 25, and 125 μmol/L for 24 h Cell viability was measured by using MTT assay. AZM or AZM derivatives exhibited no cytotoxicity at any tested concentration as compared to unstimulated, nontreated BMDM control (dotted line). Moreover, AZM and AZM derivatives at 25 and/or 125 μmol/L significantly increased proliferation of BMDMs as compared to unstimulated controls at *P < 0.05, **P < 0.01, ***P < 0.001. Data are mean ±SD and representative of three independent biological replicate experiments

We chose to use primary bone marrow‐derived macrophages (BMDMs) for our studies as BMDM responses in vitro are predictive of CNS macrophage response in vivo. When stimulated with LPS+IFN‐gamma), BMDMs model proinflammatory macrophages found in neuropathologies.^2^, ^8^, ^19^ No doses of AZM or its derivatives were toxic to BMDMs when applied directly to the cells for 24 hours (Figure 1). Interestingly, this prolonged stimulation of BMDMs with high doses of AZM and its derivatives resulted in increased readouts on the MTT assay indicative of increased BMDM proliferation or increased metabolic activity (Figure 1). This effect was not as robust after 6 hours of stimulation (Figure S8), and therefore, a 6‐hr stimulation time point was used for subsequent assays.

### 3.2 Macrophage IL‐12/IL‐10 levels with derivatives

**Figure 2:**
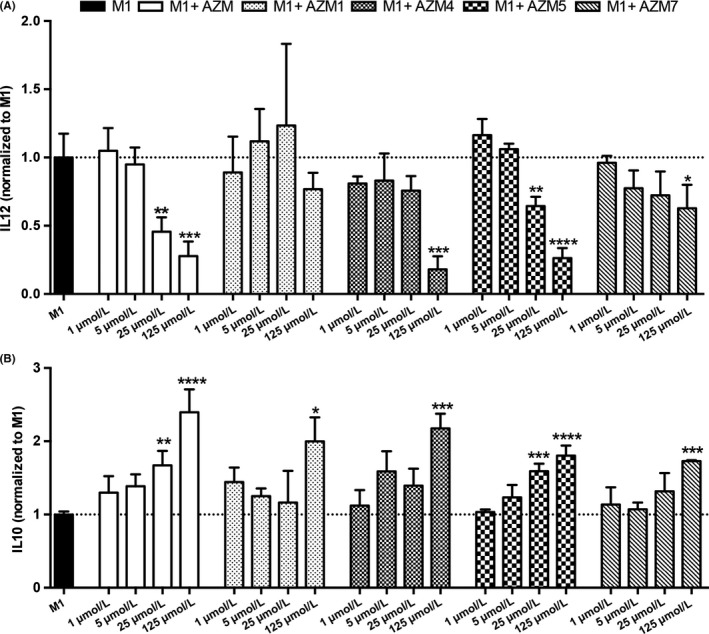
Nonantibiotic macrolides polarize proinflammatory macrophages to an anti‐inflammatory phenotype. BMDMs were polarized to be M1 macrophages by stimulating with LPS +INF‐gamma. AZM, AZM1, AZM4, AZM5, and AZM7 were coapplied to M1 cells at concentrations of 1, 5, 25, and 125 μmol/L for 6 h Protein levels of IL‐12 (A) and IL‐10 (B) in cell culture medium were analyzed by ELISA and expressed as fold change over M1 of mean ± SD. *P < 0.05, **P < 0.01, ***P < 0.001 vs M1. Data are representative of three independent biological replicate experiments. Each experiment was performed in triplicates per treatment group. (A) AZM and AZM 5 at the concentrations of 25 and 125 μmol/L significantly decreased proinflammatory cytokine IL‐12, while AZM 4 and AZM 7 significantly reduced IL‐12 level only at the concentration of 125 μmol/L. AZM 1 showed no effect in changing IL‐12 secretion. (B) The anti‐inflammatory cytokine IL‐10 level was significantly increased in M1 macrophages coincubated with AZM and AZM 5 at the concentrations of 25 and 125 μmol/L; while AZM 1, AZM 4, and AZM 7 significantly increased IL‐10 expression only at the highest tested concentration of 125 μmol/L (B)

The relative expression of IL‐10 and IL‐12 is a defining feature of M1 and M2 macrophages^27^ with M2 macrophages producing high levels of IL 10 and low levels of IL‐12. Conversely, M1 macrophages produce substantial amounts of IL‐12 and minimal IL‐10. These cytokine profiles are also predictive of the neurotoxic potential of stimulated macrophages with toxic potential decreasing with increased IL‐10 and reduced IL‐12 production.^7^, ^28^ Similar to previous observations,^6^, ^7^ AZM reduced production of the proinflammatory cytokine, IL‐12, and elevated the secretion of the anti‐inflammatory cytokine IL‐10 in M1 macrophages in a dose‐dependent manner 6 hours after stimulation. Compared to M1‐stimulated macrophages, costimulation of M1 stimulant (LPS +IFN‐gamma) with either 25 or 125 μmol/L AZM significantly decreased IL‐12 (*P* < 0.01 and <0.001, respectively) and increased IL‐10 (*P* < 0.01 and <0.0001, respectively; Figure 2).

To determine the immunomodulatory properties of our azithromycin derivatives, we examined IL‐10 and IL‐12 production in BMDMs costimulated with derivatives and M1 stimulants (LPS + INF‐gamma). We detected significantly decreased IL‐12 production with 125 μmol/L cotreatment concentrations for derivatives 4 (*P* < 0.001) and 7 (*P* < 0.05) relative to M1 stimulation alone (Figure 2). Derivative 5 had significantly reduced IL‐12 production with 25 μmol/L (*P* < 0.01) and 125 μmol/L (*P* < 0.0001) concentrations. There was no significant effect on IL‐12 with derivative 1. We observed reciprocal significant increases in IL‐10 with all derivatives at the highest dose of 125 μmol/L (*P* < 0.05, Figure 2). In addition, the 25 μmol/L stimulation with derivative 5 significantly increased production of IL‐10 relative to M1 (*P* < 0.001; Figure 2). RT‐PCR analyses of select genes associated with M1 or M2 macrophage phenotypes (ie, IL‐6, IL‐1b, TNF‐a, and TGF‐b) demonstrated similar immunomodulatory effects between AZM and derivatives 4 and 7 (Figure S9). Collectively, these data demonstrate that altering the bacterial binding residues of AZM does not reduce its immunomodulatory properties with AZM5 having similar dose‐response properties as the parent compound.

### 3.3 Macrophage‐mediated neurotoxicity with derivatives

**Figure 3:**
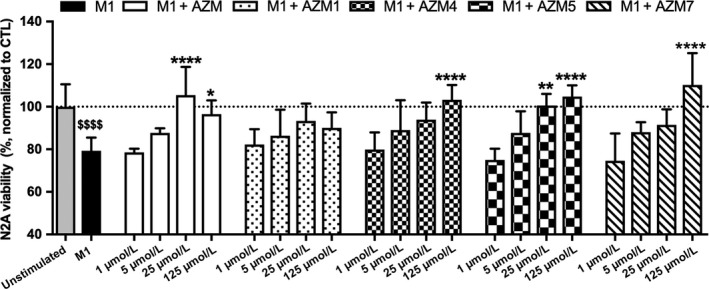
Nonantibiotic macrolides decrease the neurotoxic potential of proinflammatory macrophages. Neuron 2A (N2A) cells were incubated with MCM for 24 h MCM from M1 macrophages reduced neuron cell viability significantly as compared to control (N2a cells treated with MCM from unstimulated, nontreated BMDMs, dotted line), M1 vs Control at $$$$ *P* < 0.0001). MCM from M1 macrophages coincubated with AZM and/or AZM 4, AZM 5, and AZM 7 at 25 and/or 125 μmol/L restored neuron cell viability as compared to M1 MCM (*P < 0.05, *P* < 0.01, *P* < 0.0001 vs M1). Derivative AZM 1 exhibited no effect in modulating M1 MCM‐induced neurotoxicity. Data are mean ± SD and representative of three independent biological replicate experiments

We previously reported that M1 supernatant, that is, macrophage‐conditioned medium (MCM), is neurotoxic when applied to neuron cells and treatment of M1 macrophages with AZM significantly alleviates this neurotoxic effect.^2^, ^7^ Since we have observed similar effects of M1 MCM on both primary neurons and the neuro2A cell line,^2^, ^7^ we chose to evaluate the neurotoxic potential of AZM and derivatives using neuro2a (N2A) cells. Consistent with these previous observations, M1 MCM significantly reduced N2A cell viability (*P* < 0.0001 vs N2A cells incubated with unstimulated/untreated MCM; Figure 3). MCM generated by BMDM costimulation with LPS + INF‐gamma and AZM (25 and 125 μmol/L) significantly reversed the MCM toxicity (25 μmol/L *P* < 0.0001; 125 μmol/L *P* < 0.05 vs M1; Figure 3). Direct application of AZM on N2A cells had no effect on cell viability, and MCM isolated from BMDMs treated with AZM in the absence of M1 stimulants had no effect on neuron viability (Figure 3).

AZM5 was the most comparable to AZM in alleviating neurotoxicity of M1‐stimulated BMDMs, with concentrations of 25 and 125 μmol/L both showing significant neuroprotective effects (*P* < 0.01 and *P* < 0.0001, respectively, vs M1). Derivatives AZM4 and AZM7 at the concentration of 125 μmol/L also significantly attenuated the neurotoxic effect of M1 MCM (*P* < 0.0001 vs M1). Contrastingly, AZM1 did not significantly decrease M1 MCM‐induced neurotoxicity at any tested concentrations.

## 4 DISCUSSION

In this study, we demonstrate the retention of immunomodulatory activity in AZM derivatives with altered sugar moieties using ourin vitro model of macrophage CNS inflammation. This model accurately predicts the macrophage/microglial response in the injured CNS.^7^, ^8^, ^19^, ^20^ Specifically, we demonstrate that these derivatives, like the AZM parent compound, have no negative toxic effects on macrophage viability, retain the ability to polarize M1 macrophages toward the M2 phenotype as determined by IL‐10/12 cytokine profiles, and are equally effective in reducing the neurotoxic effects of M1 macrophage supernatants on neuronal cultures. In these studies, the ability of the derivatives to shift IL‐10/12 cytokine profiles closely correlated with their ability to mitigate M1 supernatant toxicity to neurons. This supports our notion of utilizing IL‐10/12 cytokine profiles as an indicator of M1/M2 macrophage polarization and neurotoxicity. Recent literature demonstrates that AZM increases reparative macrophage activation in rodent models of spinal cord injury, stroke, lung infection, skin inflammation, and in humans with cystic fibrosis.^7^, ^8^, ^9^, ^10^, ^11^, ^12^, ^29^, ^30^, ^31^ This anti‐inflammatory mechanism, potentially unrelated to AZM's antibacterial properties, holds great promise in the treatment of these diverse inflammatory conditions.

Further, this relatively unexplored therapeutic approach could likely be exploited more effectively with continued optimization of therapeutics such as AZM and related macrolides. In particular, one major obstacle in the clinical development of anti‐inflammatory macrolide antibiotics, such as AZM, is the concern that increased use of these drugs for their secondary anti‐inflammatory effects may inadvertently promote bacterial resistance to this antibiotic in the treatment of a variety of infections. In the spinal cord injury patient population, for example, AZM is the antibiotic of choice for treating recurrent respiratory infections and pneumonia,^32^ a leading cause of death following spinal cord injury; thus, antibiotic resistance is a major concern. Fortunately, recent studies have indicated that macrolides modified to remove their antibacterial activity retain beneficial anti‐inflammatory effects in models of inflammatory skin disorders and chronic lung diseases.^13^, ^16^

Collectively, we demonstrate that AZM derivatives with altered sugar moieties retain immunomodulatory properties. We did not, however, observe uniform immunomodulatory and neuroprotective properties with all derivatives tested. AZM7, which had the most extensive chemical modifications (diacetylation and the removal of the cladinose moiety), invoked modest immunomodulatory effects exclusively at the highest concentration tested. Interestingly, however, AZM7 did not retain any antistaphylococcal activity. Similarly, AZM4, which also lacks the cladinose that is replaced by a carbonyl, had modest yet significant immunomodulatory effects with minimal antistaphylococcal activity. Although AZM1 at the concentration of 125 μmol/L significantly increases IL‐10 level, it has no effect in reducing IL‐12 production. Interestingly, AZM1 also induced significant but small changes, relative to AZM, in BMDM metabolic activity at this high dose. AZM5, which was the only derivative tested without the cladinose removed, closely mimicked or slightly exceeded AZM's activity at all concentrations tested including the BMDM MTT assay. Unfortunately, the acetylation of both sugars in AZM5 did not abolish the antistaphylococcal activity as desired. This may suggest, however, that chemical modifications to substitute the cladinose with functional variants may be an effective approach in developing subsequent generations of derivatives. Future studies that systematically alter each of these components may better clarify which of these modifications are beneficial or detrimental in retaining/improving AZM's immunomodulatory activity.

We demonstrated the clear anti‐inflammatory activity of these AZM derivativesin vitro*;* however, related studies utilizing derivatives of AZM and other macrolides suggest that these drug candidates likely hold great potential for treating inflammatory disorders of the CNS in vivo*.* For example, Sugawara et. al (2012) developed a series of anti‐inflammatory nonantibiotic macrolide derivatives in vitro and then successfully used these derivatives in an in vivo model of inflammatory bowel disease.^14^ Together with our prior work demonstrating the predictive nature of our in vitro model,^7^, ^8^, ^19^, ^20^ these results suggest that our compounds hold great promise in treating the detrimental neuroinflammatory conditions. Given that there are extremely few treatment options for most neurological disorders, our current findings clearly demonstrate the importance of these drugs and support their continued development into novel therapeutics to treat CNS inflammation.

While the current study and related publications show encouraging therapeutic outcomes following stimulations with derivatives of AZM and other macrolides, the exact mechanisms of action remain uncertain. Much of the work in this regard has converged in identifying the NF‐κB signaling cascade as the core regulator of the observed shifts in cytokine profiles following macrolide treatment.^29^, ^33^, ^34^, ^35^ While this is clearly important, the molecular target upstream of the NF‐κB cascade on which these drugs act remains unclear.^36^ In vitro studies show that AZM accumulates in macrophage lysosomes, where it increases the pH, interacts with membrane lipids, induces phospholipidosis, and alters vesicular trafficking which may affect endocytosis and phagocytosis.^37^, ^38^ Other suggested mechanisms indicate that AZM may alter cellular autophagy, or alter the TLR4 signaling pathways by changing endosome trafficking.^37^ Further, it remains unknown whether these findings remain valid in vivo or whether derivatives of AZM retain the same mechanism of action. While complicated, continued work in these areas is essential as it could lead to new therapeutics, such as the compounds described here, or provide novel therapeutic targets for future drug development.

One interesting finding in the current study was the fairly pronounced increase in macrophage viability when treated with the highest dose of AZM or derivative for 24 hours (widely used time point for measuring drug toxicity in vitro). This assay measures the conversion of tetrazolium dye MTT 3‐(4,5‐dimethylthiazol‐2‐yl)‐2,5‐diphenyltetrazolium bromide to formazan by NAD(P)H‐dependent cellular oxidoreductase enzymes, and this measure of metabolic activity is routinely used to quantify changes in cell number or vitality. How AZM and its derivatives induce this effect at high concentrations, and how this may alter inflammatory activities remains unclear, however, it is unlikely that this is directly related to our observed shifts in cytokine profiles and neurotoxicity across drug concentrations. If the observed increases in IL‐10 following AZM stimulations were simply a result of cellular proliferation, then IL‐12 would also be expected to rise, instead, however, IL‐12 levels fell dramatically. Similarly, measurements of cellular proliferation/metabolism at the 6‐hour time point, when we measure IL‐10/12 levels, displayed more modest increases in proliferation/metabolism and are thus less likely to influence our cytokine profiles.

In conclusion, we have identified AZM derivatives that retain key immunomodulatory functions in our in vitro model of CNS inflammation. While the antiinfective properties of the derivatives were associated with neuroprotection, we also observed that some derivatives with greatly reduced antiinfective characteristics retained neuroprotective and anti‐inflammatory functions. Although a limited sample size of derivatives was created and tested, this indicates that the antibiotic properties of AZM may not be required for immunomodulatory‐mediated neuroprotection. With continued development, these compounds could become viable clinical neuroprotectants and immunomodulatory treatments for neuropathologies. Additionally, given the usage of AZM's anti‐inflammatory properties across disciplines, these drugs hold great potential in treating a wide variety of inflammation‐based human disorders.

## CONFLICT OF INTEREST

The authors declare no conflict of interest.
