# Supplementary material for: Macrolide derivatives reduce proinflammatory macrophage activation and macrophage‐mediated neurotoxicity
Source: CNS Neurosci Ther. 2019 Jan 24;25(5):591–600. doi: 10.1111/cns.13092 (PMC6488883; doi:10.1111/cns.13092)
Supplement: Supplementary file 1 [file CNS-25-591-s001.pdf]

SUPPORTING INFORMATION

Title: **Macrolide derivatives reduce pro-inflammatory macrophage activation and macrophage-mediated neurotoxicity.**

Short Running Title: **Macrolides reduce macrophage neurotoxicity**

Bei Zhang[1], Timothy J. Kopper[1], Xiaodong Liu[2], Zheng Cui[2], Steven G. Van Lanen[2*], John C. Gensel[1*]

Author's Address:

[1]Spinal Cord and Brain Injury Research Center

Department of Physiology

College of Medicine

[2]Division of Bioorganic, Medicinal, & Computational Chemistry,

Department of Pharmaceutical Science

College of Pharmacy

University of Kentucky

Lexington, KY 40536, United States.

***Correspondence to:**

John C. Gensel

B463 Biomedical & Biological Sciences Research Building (BBSRB)

University of Kentucky

741 S. Limestone Street

Lexington, KY 40536-0509

(859) 218-0516

gensel.1@uky.edu

or

Steven Van Lanen

321 TODD Building

789 South Limestone Street

Lexington, KY 40536-0596

Phone: (859) 323-6271

svanlanen@uky.edu

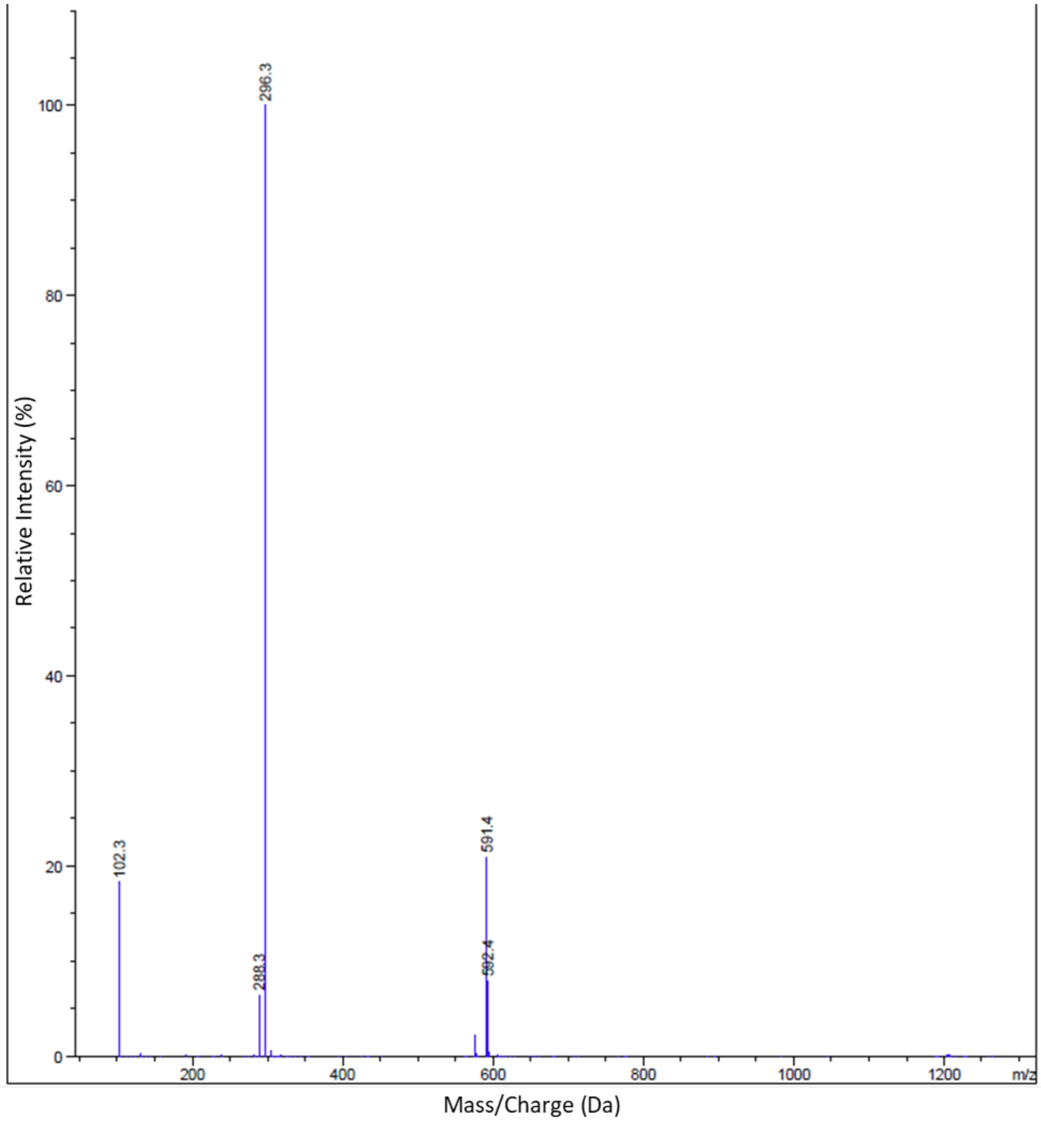

Figure S1. Mass spectrum of AZM1. MS (ESI) *m/z*: $[M + H]^+$ calcd for $C_{30}H_{59}N_2O_9$ 591.4; found 591.4.

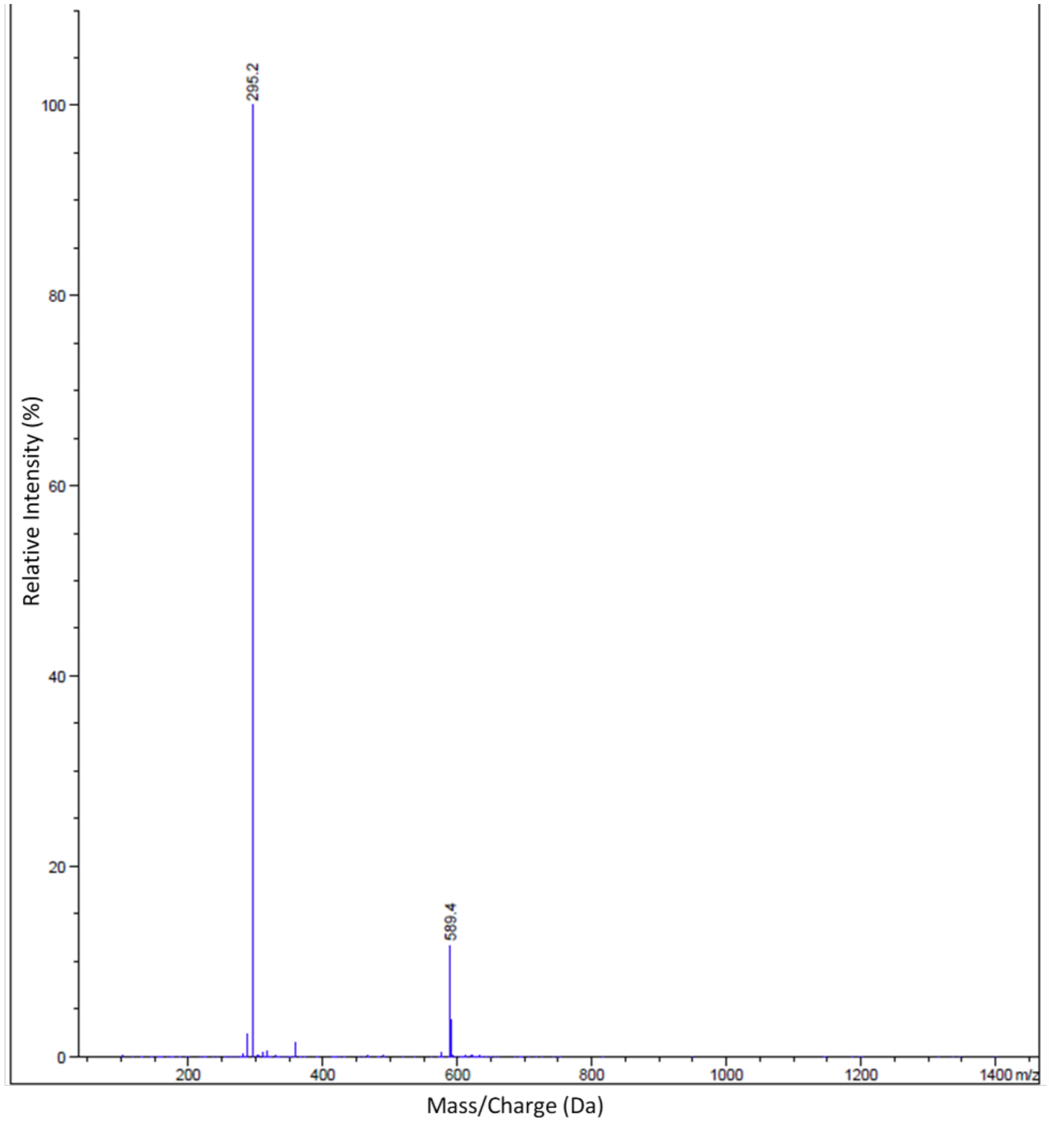

Figure S2. Mass spectrum of AZM4. MS (ESI) *m/z*: $[M + H]^+$ calcd for $C_{30}H_{57}N_2O_9$ 589.4; found 589.4.

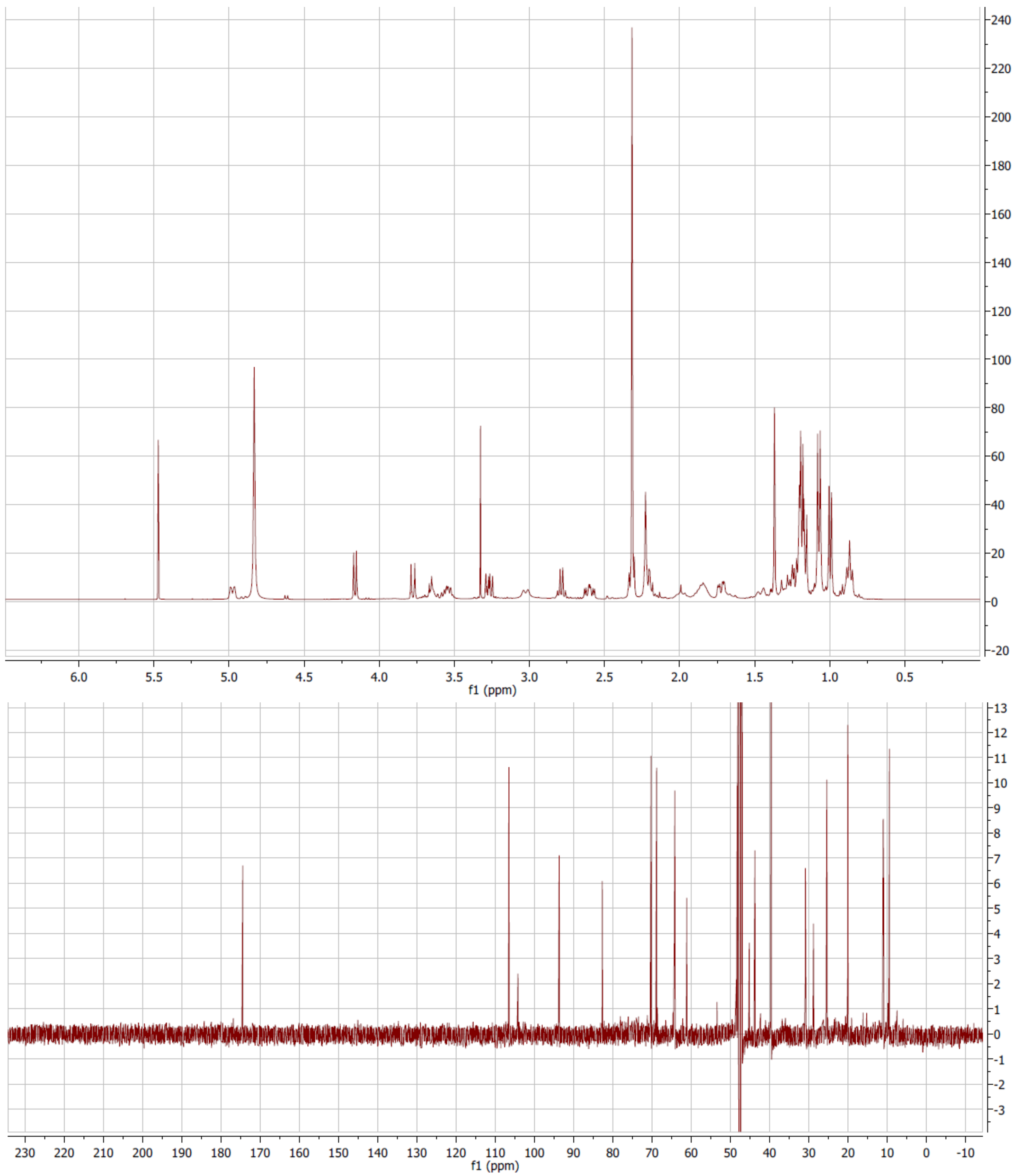

Figure S3. ^{1}H NMR spectrum (D_2O, 400 MHz) and ^{13}C NMR spectrum (D_2O, 101 MHz) of AZM4.

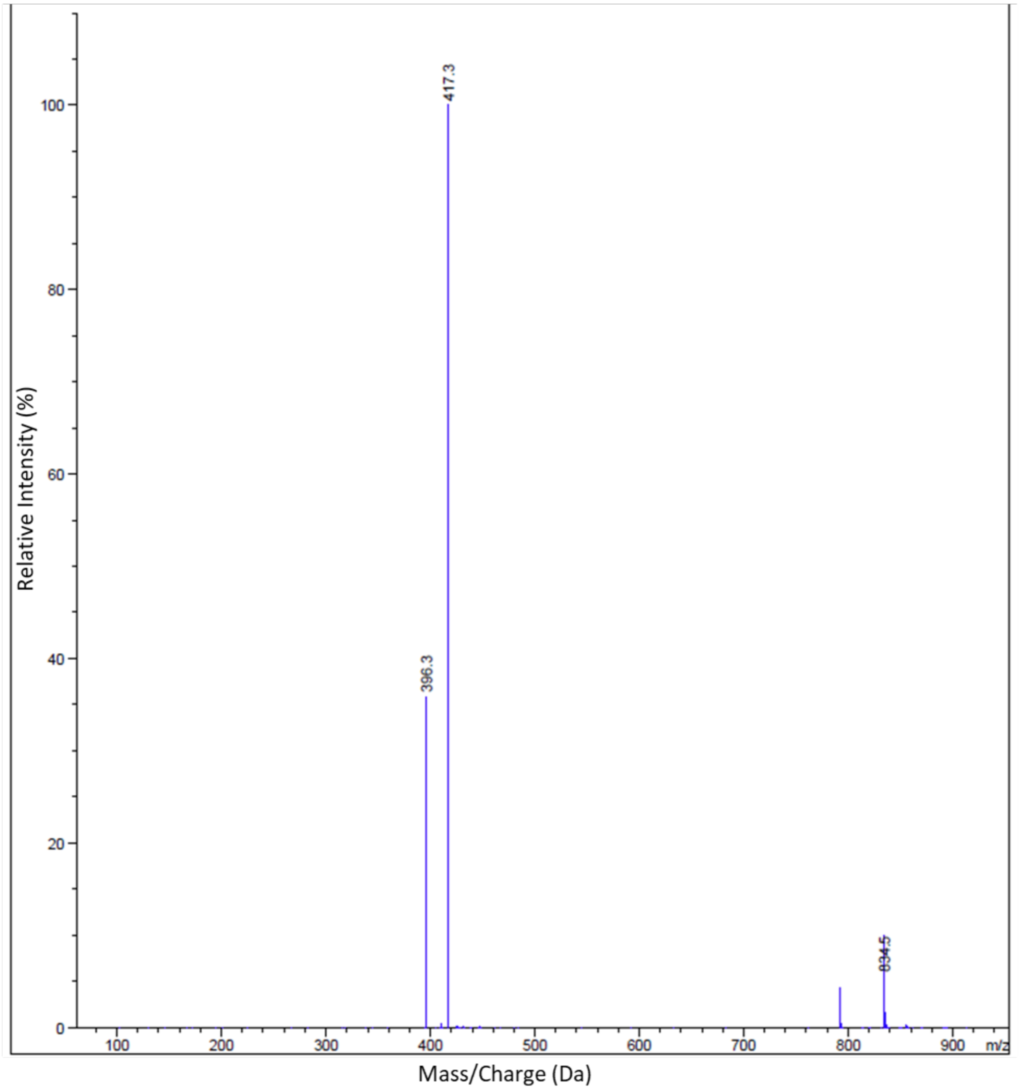

Figure S4. Mass spectrum of **AZM5**. MS (ESI) *m/z*: $[M + H]^+$ calcd for $C_{42}H_{77}N_2O_{14}$ 834.5, found 834.5.

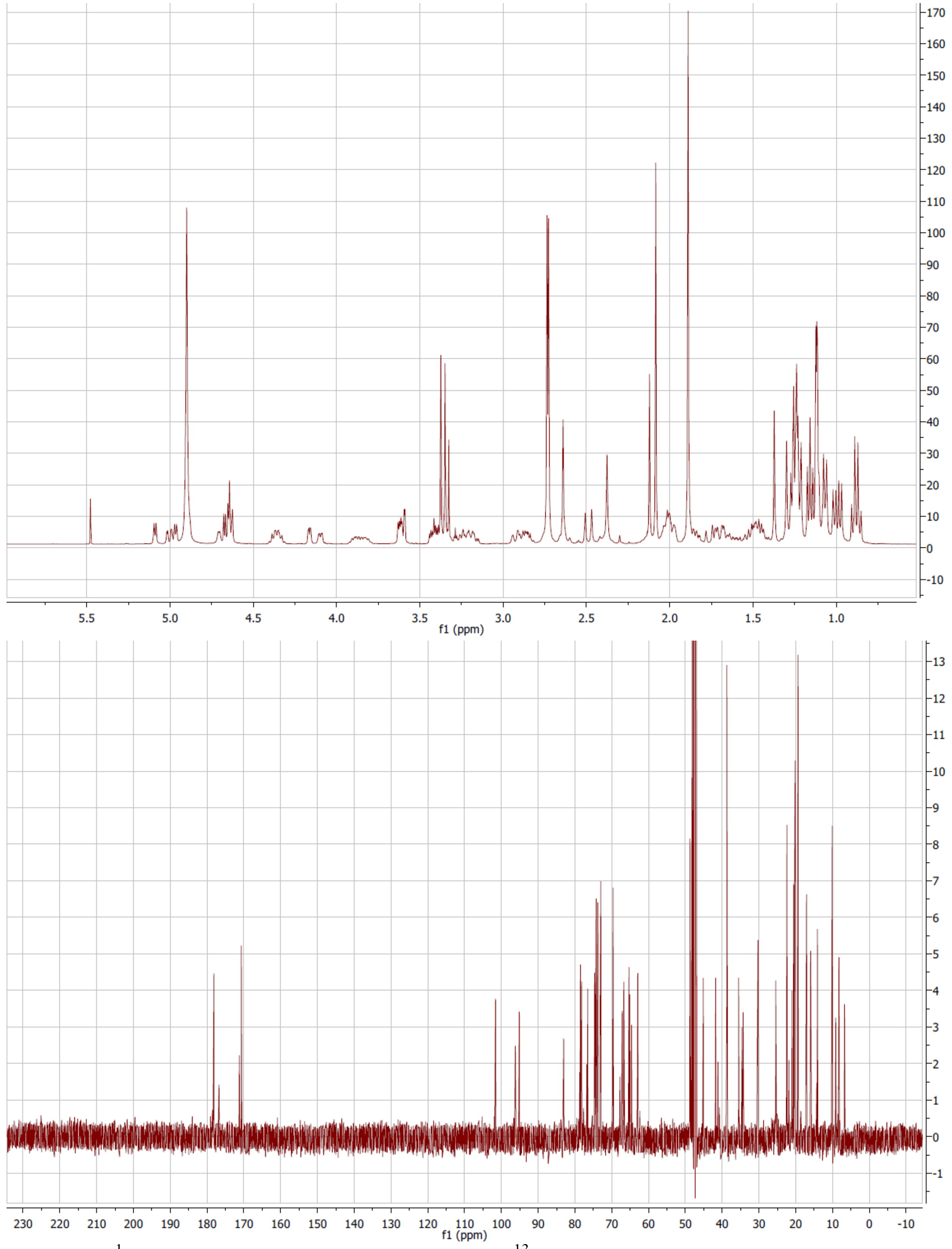

Figure S5. ^{1}H NMR spectrum (D_2O, 400 MHz) and ^{13}C NMR spectrum (D_2O, 101 MHz) of AZM5.

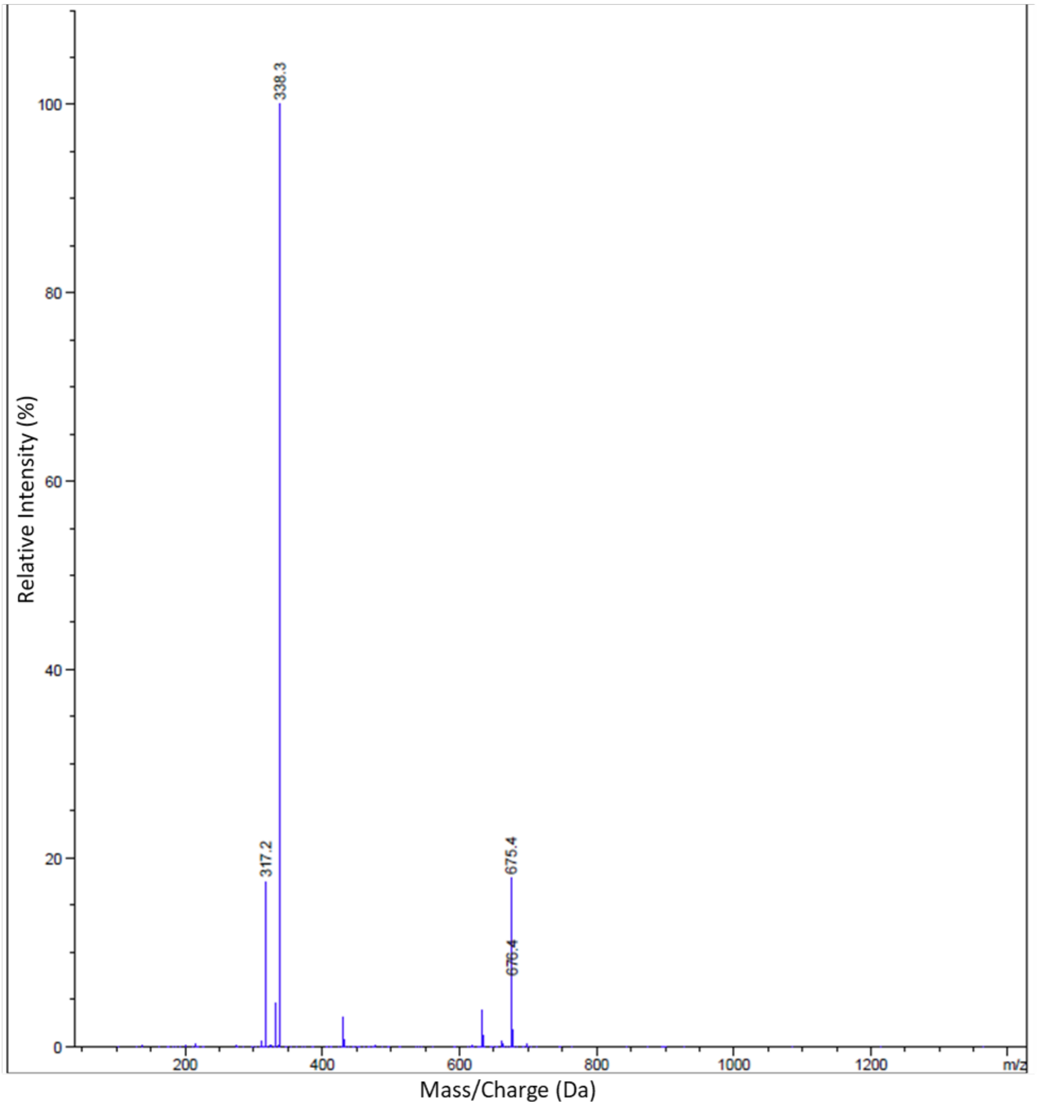

Figure S6. Mass spectrum of AZM7. MS (ESI) *m/z*: $[M + H]^+$ calcd for $C_{34}H_{63}N_2O_{11}$ 675.4, found 675.4.

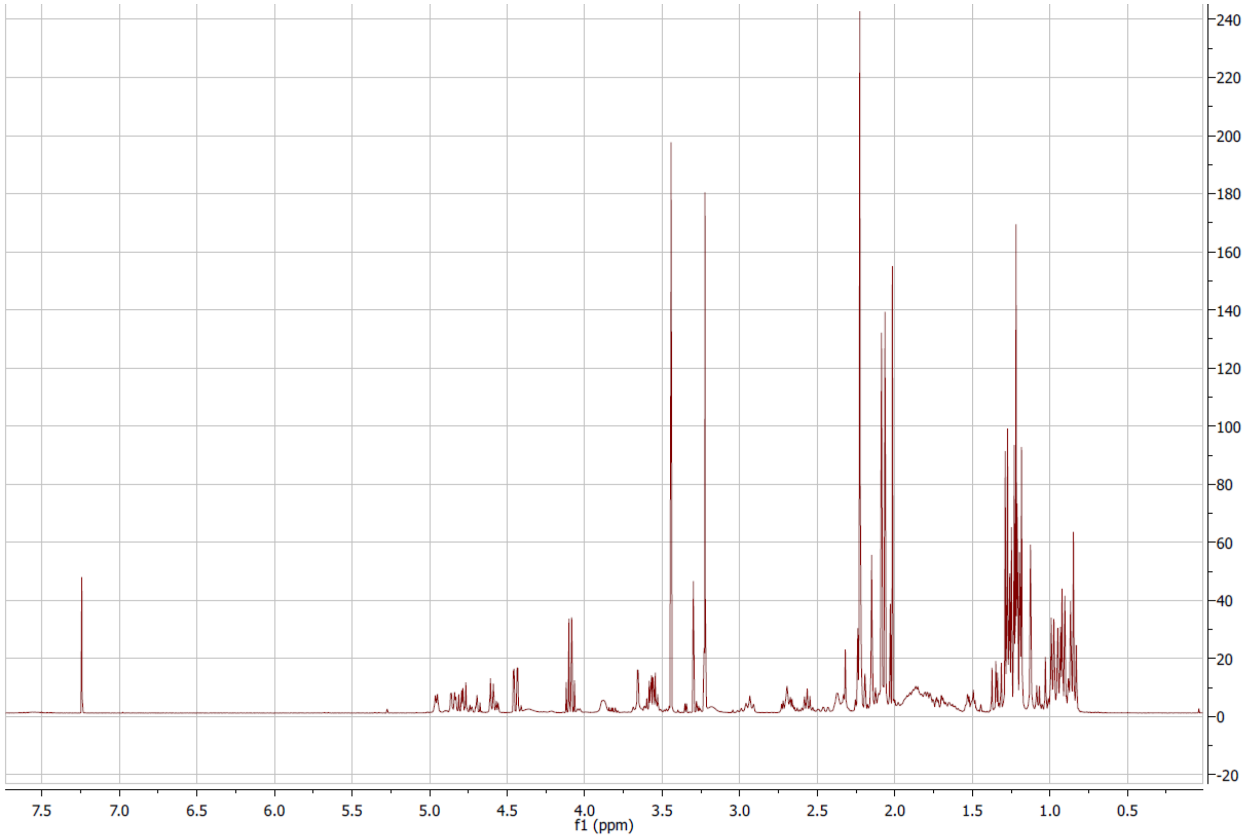

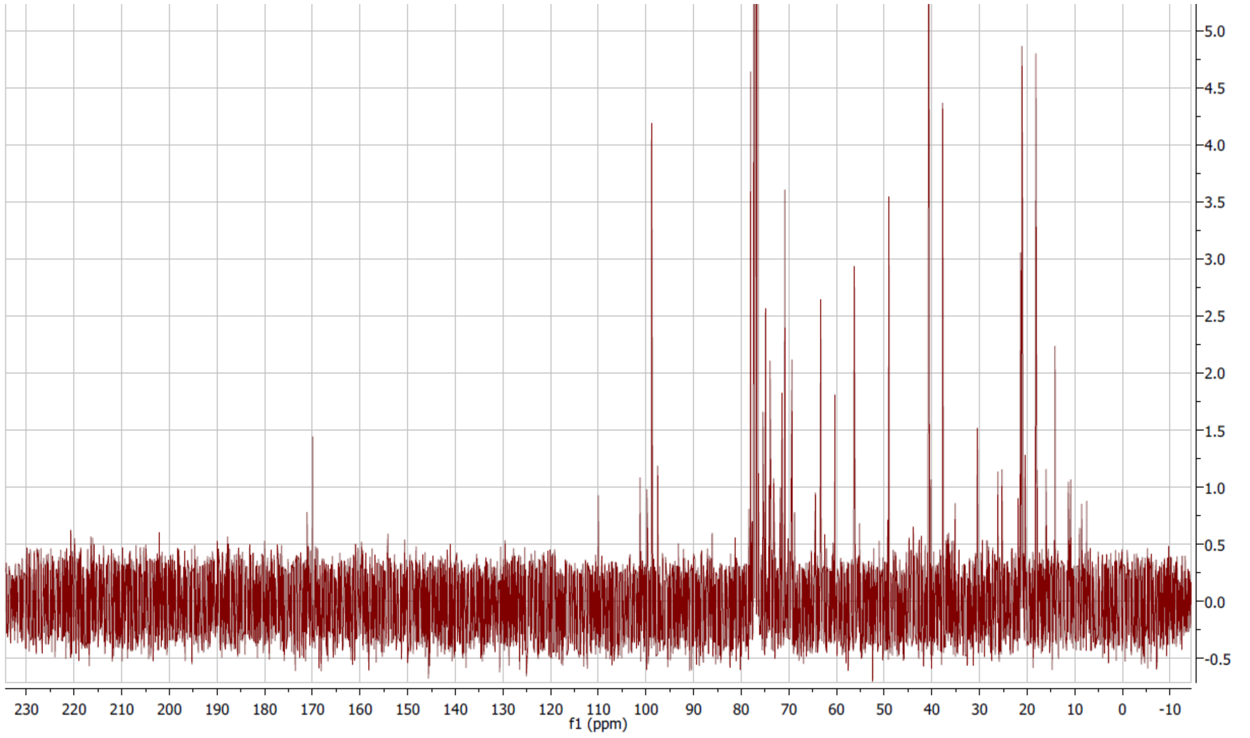

Figure S7. ^{1}H NMR spectrum (D_2O, 400 MHz) and ^{13}C NMR spectrum (D_2O, 101 MHz) of AZM7.

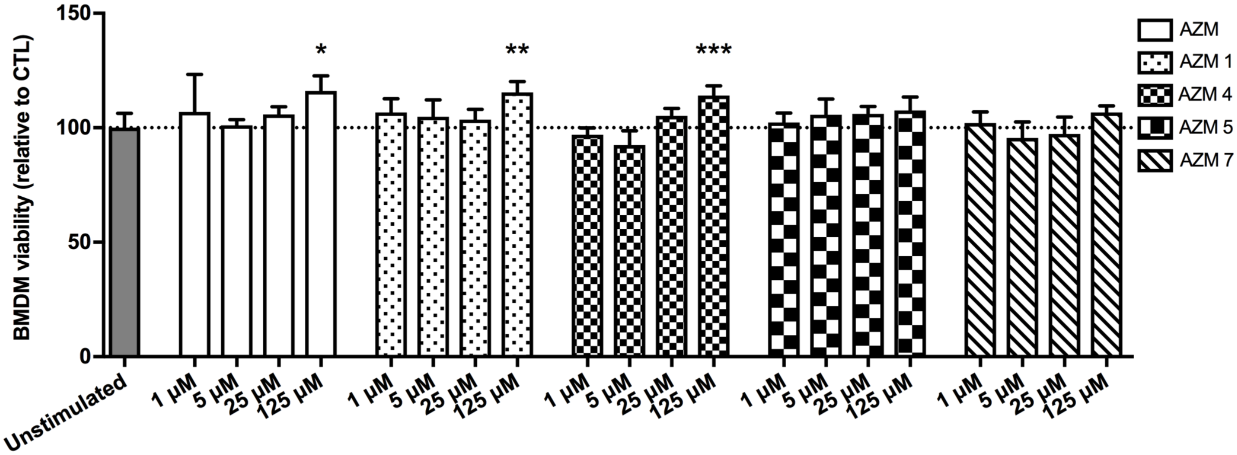

Figure S8: Altering the antibiotic properties of AZM does not decrease macrophage viability at the time of protein and RNA isolation. Bone marrow-derived macrophages (BMDMs) were isolated from adult mice and were treated with AZM, AZM1, AZM4, AZM5, and AZM7 at concentrations of 1, 5, 25, and 125 μM for 6 hrs (timepoint at which samples are collected for protein, RNA, and neurotoxicity assays). Cell viability was measured by using MTT assay. AZM or AZM derivatives exhibited no cytotoxicity at any tested concentration as compared to unstimulated, non-treated BMDM control (dotted line). Moreover, AZM and AZM derivatives 1, and, 4 at 125 μM significantly increased proliferation of BMDMs as compared to unstimulated controls at *$p<0.05$, **$p < 0.01$, ***$p < 0.001$, however this effect was less robust than the 24hr drug toxicity timepoint in Fig. 2. Data is mean ± SD and representative of three independent biological replicate experiments.

Methods: Performed as in figure 2, utilizing a 6hr timepoint.

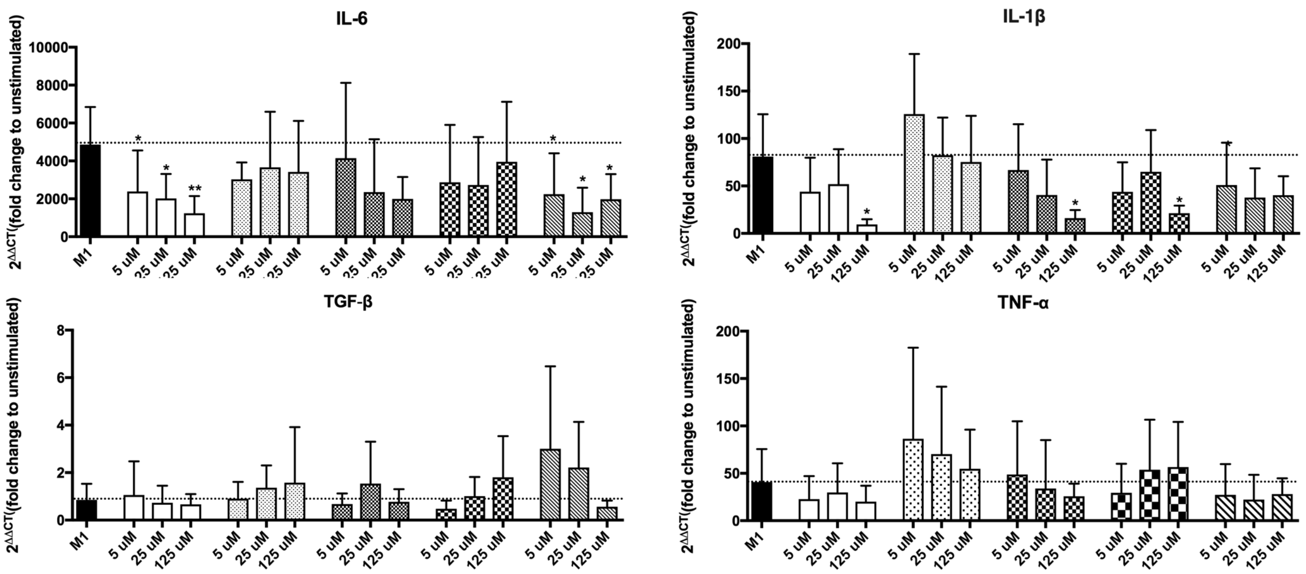

Figure S9: AZM and AZM derivatives decrease pro-inflammatory macrophage activation. The pro-inflammatory cytokine, IL-1-beta, was down regulated by AZM, AZM 4, and AZM 7. Similarly the pro-inflammatory cytokine, IL-6 was, down regulated by AZM and AZM 7. Shifts in the anti-inflammatory cytokine TGF-Beta and the pro-inflammatory cytokine TNF-alpha were not statistically significant. Results indicative of 2 independent biological replications. *$p<0.05$, **$p < 0.01$, ***$p < 0.001$

Methods: BMDMs were polarized to be M1 macrophages by stimulating with LPS + IFN-gamma. AZM, AZM1, AZM4, AZM5, and AZM7 were co-applied to M1 cells at concentrations of 1, 5, 25, and 125 μM for 6 hr. 300μL TRIzol LS reagent (Life Technologies) was added to each well (500,000 cells) to isolate RNA. Total RNA was isolated based on the manufacturer's protocol, with an additional phase separation using BCP, precipitation with isopropanol (Sigma-Aldrich, St. Louis, MO), and wash of the isolated RNA in 70 % ethanol. Then, 1 μg RNA was reverse-transcribed using the high- capacity complementary (cDNA) reverse transcription kit (Life Technologies). Real-time PCR amplification was performed on the mixture of 100 ng cDNA sample, Taqman Universal PCR Master Mix, and Taqman Probes (Life Technologies) using the Applied Biosystems Step One Plus Real-Time PCR System. Expression of genes was normalized to 18S mRNA for each sample, and reported values were calculated as $2^{-\Delta\Delta CT}$.
